# Congress Report on the Discovery of the Letheon

**Published:** 1849-06

**Authors:** 


### CONGRESS REPORT ON THE DISCOVERY OF THE LETHEON.

A majority of the committee, to whom was referred the memorial of William T. G. Morton, asking compensation from Congress for the discovery of the anæsthetic property of sulphuric ether, have reported in favor of the claims of the memorialist. Dr. Edwards, the chairman of the committee, has made out a strong case for Dr. Morton; but the minority report is said to place the claims of Dr. Jackson in a favorable light. We have not yet received a copy of the latter report, and have not leisure to enter into the merits of the controversy even if we had all the documents before us.

